# The effect of induced optimism on the optimistic update bias

**DOI:** 10.1186/s40359-020-0389-6

**Published:** 2020-03-29

**Authors:** Shinpei Yoshimura, Yuma Hashimoto

**Affiliations:** Department of Psychology, Otemon Gakuin University, 2-1-15, Nishi-ai, Osaka, Ibaraki Japan

**Keywords:** Belief updating, Depression, Dysphoria, Hopelessness, Optimism

## Abstract

**Background:**

Individuals tend to have an optimism bias, processing desirable information more frequently than undesirable information. However, people who have been diagnosed with depression often have a more pessimistic view of the future. A recent study suggests that future expectations in individuals with dysphoria become more optimistic when asked to make optimistic future predictions about the future. In the present study, we investigated the differential effects of induced optimism training which making optimistic future prediction to change future beliefs in normal participants with various levels of dysphoria (low, mild, high).

**Methods:**

We recruited normal participants (*n* = 69) from a local university students and divided participants into three groups (low, mild, high dysphoria) by measuring dysphoric mood. These three groups were assigned to the induced optimism training or control condition. After the training, participants performed the two-stage belief updating task. In the first stage, participants estimated their personal probability of experiencing adverse events while being presented with the average probability of the event occurring to a living person. This information could be desirable for participants(when presented with a probability that was below their estimation) or undesirable (when presented with a probability that was above their estimation). To assess how desirable versus undesirable information influenced beliefs, participants estimated their personal probability of experiencing the events again in the second stage. The amount of update error was calculated as the difference between the estimates in the first stage and the second stage. The difference between the errors was classified as the update bias.

**Results:**

After the induced optimism training, individuals with the mild dysphoria demonstrated a higher update bias than low (*p* < .001) and the high dysphoria (*p* < .05) group in induced optimism condition. Significant differences were not found in control group. Results indicates that individuals in the mild dysphoria group showed an increased update bias after being exposed to the induced optimism training Dysphoric mood and trait optimism remained unchanged in both the experimental and control groups.

**Conclusions:**

Results suggest that induced optimism training has potential to change individuals with mild dysphoria perceptions’ about the future.

## Background

Optimism is defined as the tendency to overestimate future positive events and underestimate future negative events [1]. Day to day, we have optimistic or pessimistic views about our future on a moment-to-moment basis. As Hecht [2] stated, “People may shift positions on the optimism-pessimism continuum as time unfolds”. However, individuals with depression often expect negative future events to occur more often. The cognitive model of depression has suggested that excessive estimation of future negative events is a core feature of depression [3]. For example, the hopelessness theory of depression suggests that depression is characterized by negative expectations that desired outcomes will never occur and that one’s own behavior is not effective in achieving desired outcomes [4]. Seminal work by Seligman [5] suggests learned helplessness, the belief that one does not have control over the environment, can induce depression. Seligman argued that a pessimistic perspective is associated with depression, and an optimistic perspective is associated with lower levels of depression. Other evidence suggests that individuals with depression do in fact hold more pessimistic expectations about the future than do individuals without depression, perceiving positive events as less likely and negative events as more likely [6–8]. Based on these findings, individuals with depression are considered to have a negative outlook about the future. Additionally, individuals with dysphoria can maintain a negative outlook about the future as well. In the literature, the term dysphoria has been used to refer to subclinical populations with elevated depression scores who do not meet the diagnostic criteria for major depression (see Kendall, Hollon, Beck, Hammen, & Ingram [9]. Dysphoria is an emotional states which includes discontent, and unhappiness [10]. Dysphoria can be considered as subclinical levels of depression [11], and analogues of clinical symptom of depression [12].

Several studies also indicate that individuals with depression or dysphoria show a reduced positive outlook about the future but not an overestimated negative outlook [13–15]. In other words, individuals with depression do not necessarily have increased negative views of the future. In a recent study, Garret & Sharot [16] reported that people alter their beliefs to a greater extent in response to good news versus bad news. This asymmetry can lead to a positive bias in beliefs regarding one’s future, referred to as unrealistic optimism. In their study, participants were asked to estimate the probability of experiencing negative future events in two sessions. Between the first and second sessions, participants received the actual probability of negative events occurring in general population. If the actual probability was lower than their estimation, the information was positive, and if it was more than their estimation, it was viewed as negative. In the second session, participants adjusted their estimation of the probability experiencing negative future events. The optimism bias was defined as the difference between participants’ perceptions after being confronted with desirable and undesirable information, respectively. That is to say, optimism might be not simply just thinking positively. The optimism bias is also defined as the difference between a person’s expectation and the outcome that follows. If expectations are better than reality (either positive or negative), the bias is optimistic. Interestingly, Korn, Sharot, Walter, Heekeren, & Dolan [17] demonstrated that when faced with desirable or undesirable information regarding future events, patients with depression showed unbiased beliefs compared to normal healthy participants.

The question remains if experimental manipulation can change the optimism bias. A recent meta-analysis [18] investigated the effects of psychological intervention to increase optimism and found a moderate effect size (*g* = .41). Meevissen, Peters, & Alberts [19] reported increased optimism and positive future expectations after 2 weeks of intervention. Miranda, Weierich, Khait, Jurska, & Andersen [20] conducted a similar study and found that dysphoric participants in the mental rehearsal condition increased optimistic predictions compared to those in the control condition, and participants who had moderate depressive mood also showed a decrease in pessimism about future events. Results of these studies suggest that experimental manipulation can affect one’s optimistic view of the future even with depressive symptoms. However, it is unknown whether experimental manipulation can change expectations about future negative events in those with dysphoria. If optimism training can increase expectations about future positive events or decrease expectations about future negative events, it may be beneficial in the treatment of depression.

The purpose of this study is to examine the differential effects of induced optimism manipulation on beliefs about the future in individuals with varying levels of dysphoria (low, mild, high). In general, interventions aimed at increasing optimism are considered a positive psychological practice [18]. However, such interventions have a small effect size (*g* = .24) as measured by LOT-R, but a moderate effect size (*g* = .68) with other expectancy measures. Lyubomirsky, Dickerhoof, Boehm, & Sheldon [21] also suggested that positive psychological practices can relieve symptoms in mild to moderately depressed individuals. Based on these previous studies, we hypothesize that participants with mild dysphoria who received induced optimism training will show an increased optimism bias compared to individuals with high dysphoria. We predicted participants with low dysphoria will not show changes in the optimism bias compared to participants with mild and high dysphoria.

## Method

### Participants

Sixty-nine participants recruited from Otemon Gakuin University (age: mean = 19.7, SD = 1.2, range 18–23; 57 female) participated in the study. Participant’s dysphoria levels were measured with the Center for Epidemiologic Studies Depression scale (CES-D [22]). Numerous studies, which include a wide variety of participants, have supported the cutoff score of 24 to determine moderate-severe depression [23]. The traditional cutoff score of 16 to indicate mild depression has been questioned [24]. Thus, we operationally defined participant’s dysphoria level based on cumulative frequency of their CES-D score. Participants were divided into three groups (CES-D: 0–14 (low dysphoria), 15–21 (mild dysphoria), and 24-above (high dysphoria)), they were randomly assigned to two manipulation conditions.

### Measures

To measure the levels of participant’s dysphoria, the CES-D [22] was used. It is comprised of 20 items rated on a 4-point scale (0 = rarely or never and 3 = most or all the time). Higher scores indicate higher levels of depression. The internal consistency of the Japanese version of CES-D scale was confirmed, and Cronbach’s alpha was 0.84 [25].

Dispositional optimism was measured using the Japanese translation of the Life Orientation Test Revised (LOT-R [26], which reflects the extent to which individuals generally expect favorable outcomes. The LOT-R consists of 10 statements of which 3 are positively stated, 3 are negatively stated, and 4 are filler items. All items are rated along a -point continuum (0: strongly disagree – 4: strongly agree). Internal consistency of Japanese version of LOT-R was confirmed and Cronbach’s alpha was .62 [27]. In the present study, the LOT-R was used to measure effects of induced optimism on dispositional optimism.

### Manipulation conditions

The experiment consisted of two conditions. The first condition was the induced optimism condition (*n* = 30; 20 female). Participants in this condition were asked to make judgments about future positive event predictions. By using Psychopy [28], we randomly presented 69 phrases about positive events (40 phrases were highly likely to occur; 21 phrases were moderately likely to occur; 8 phrases were highly unlikely to occur) and 71 negative events (39 phrases were highly likely to occur; 24 phrases were moderately likely to occur; 8 phrases were highly likely to occur) to participants. Participants were asked to judge whether they believed the event would happen to them in the future. In the control condition (*n* = 39; 32 female), participants were presented the same phrases as in the induced optimism condition. In the control condition, participants were randomly presented 69 positive phrases and 71 negative phrases. Each phrase was comprised of the same content in the induced optimism condition. Participants in the control condition judged whether these phrases contained adjectives. Participants were randomly assigned to either the induced optimism condition or the control condition. In both conditions, participants were asked to respond as quickly as possible by pressing keys corresponding to yes/no on the keyboard. All participants engaged in these tasks across four blocks. The blocks consisted of three blocks which contained 36 phrases and 1 block which contained 32 phrases. The CES-D and life orientation test (LOT-R) [26] were measured before and after the conditions.

### Belief updating task

After the experiment, participants completed the two-stage belief updating task based on Sharot, Korn, & Dolan [29]. This was used to evaluate the cognitive bias in processing information about future events. The goal was to gather information about participants’ updating beliefs about the future in response to desirable or undesirable information, and how the updating belief might reflect the optimistic bias [17]. During the two-stage belief updating task, participants completed two sessions of negative life events estimation. In the first session, participants were presented with 40 different life events and asked to estimate their likelihood of encountering (or not encountering) each life event. All event probabilities lay between 10 and 70%. The probability of these events were based on the Statistic Bureau, Ministry of Internal Affairs and Communications in Japan [30]. Participants rated the likelihood of encountering each life event by using the keyboard. Next, participants received the actual probability of each event occurring with an average person in a similar socio-cultural environment.

According to Sharot et al. [29], the difference between participants’ estimation and actual probability of encountering life events effects is as follows. If the participant’s first estimate was higher than the average probability provided, that trial was classified as ‘desirable’ because the information presented was better than expected. Conversely, if the participant’s first estimate was lower than the average probability provided, that trial was classified as ‘undesirable’ because the information presented was worse than expected. When participants received the actual probability of each life event, if they rated the subjective probability of encountering the life event higher than actual probability in the first session, the actual probability was desirable information for the participant. But, if they rated the subjective probability of encountering the life event lower than the actual probability, received actual probability meant undesirable information. The second session was the same as the first session. However, participants were asked to estimate their likelihood of not encountering (or encountering) each life event. The instructions of the first and second sessions were counterbalanced between participants. The order of the stimuli was randomized and counterbalanced across participants. Participants’ ratings of the probability of each life event was recorded using Psychopy [28].

We preprocessed the data before analysis based on previous studies [29]. We calculated the amount of update error as the absolute difference between the estimates in first session and second session by each desirable and undesirable rating. Based on the update error of each trial, we computed the absolute average update error of both desirable and undesirable ratings for each participant. The difference between the desirable and undesirable update errors was classified as the update bias [17].

### Procedure

The Ethics Committee of Otemon Gakuin University approved the study protocol. Informed consent was obtained from all participants. First, participants completed the psychological scales including the CES-D and LOT-R. After completion they were randomly assigned to the induced optimism condition or control condition. They received instructions for each condition and participated in the training. After the training, they completed the belief updating task. Finally, they completed the same psychological scales (CES-D, LOT-R). Experimental procedure was conducted in a day. None of participants reported any discomfort during and after experiment.

## Results

### Baseline dysphoria and optimism (Table 1)

To examine the interaction between the condition and dysphoria group in baseline dysphoria and optimism, we conducted a two-way ANOVA including the condition (induced optimism/control) and the dysphoria group (low/mild/high dysphoria) with the baseline CES-D and LOT-R scores. There were no interactions among the condition and the dysphoria group on CES-D and LOT-R scores. The main effect of the dysphoria group was significant in the CES-D score *F* (2, 63) = 96.34, *p* < .001, partial *η*^*2*^ = .75, post hoc tests: high > mild > low, all *p* < .001) and LOT-R score (*F* (2,63) = 5.81, *p* < .01, partial *η*^*2*^ = 0.13, post hoc tests: low > high, *p* < .01).

**Table 1:** CES-D and LOT-R scores in each group and condition

Condition	Dysphoria group	CES-D (pre) mean(SD)	CES-D (post) mean(SD)	LOT-R (pre) mean(SD)	LOT-R (post) mean(SD)
Induced optimism	low	8.3(3.3)^*^	8.7(3.3)^*^	32.2(5.1)^*^	31.0(6.0)^*^
	mild	16.9(1.8)	14.7(4.4)	29.0(6.5)	29.7(6.0)
	high	30.5(8.2)^*^	30.4(8.2)^*^	26.3(4.4)^*^	26.3(3.8)^*^
Control	low	9.6(3.6)^**^	10.2(4.3)^**^	29.6(7.2)^**^	28.6(6.8)^**^
	mild	18.1(1.8)^**^	17.1(4.0)^**^	28.9(4.3)^**^	27.3(4.5)^**^
	high	28.9(6.9)^**^	29.9(7.0)^**^	24.5(4.8)^**^	24.7(5.2)^**^

^*^:*p* < .01, ^**^:*p* < .001

Comparison of dysphoria and optimism by the induced optimism manipulation (Table 1)

We examined change in the dysphoria (the CES-D score) and optimism (the LOT-R scores) before and after the manipulation for each dysphoria group in a three way (2 × 3 × 2) ANOVA (Condition: induced optimism vs. control x dysphoria group: low, mild, high x time: pre-manipulation vs. post manipulation). There was no significant interaction or main effect for the dysphoria and optimism conditions. The results indicate that the experiment did not affect the depressive or optimistic views of self.

### Comparison of the update bias (Fig. 1)

We sought to examine the interaction among the conditions and the dysphoria group in the update bias (difference between updates for desirable vs. undesirable information). To control for the effect of individual differences of optimism to interaction between the condition and dysphoria group, a two-way ANCOVA (including the condition and dysphoria group as between subjects factors, and the LOT-R score as the covariate) was conducted, and a significant interaction was found(*F* (2, 62) = 3.60, *p* < .05, partial *η*^*2*^ = .10). Individuals in the mild dysphoria group who participated in the induced optimism condition showed a high update bias rather than a low one (*p* < .001, *d* = 1.44) and the high dysphoria(*p* < .05, *d* = 1.13) group in induced optimism condition (both Holm’s correction). The results indicate that individuals with mild dysphoria showed an increased update bias by induced optimism manipulation. There were no significant differences in other comparisons.

**Fig. 1:**
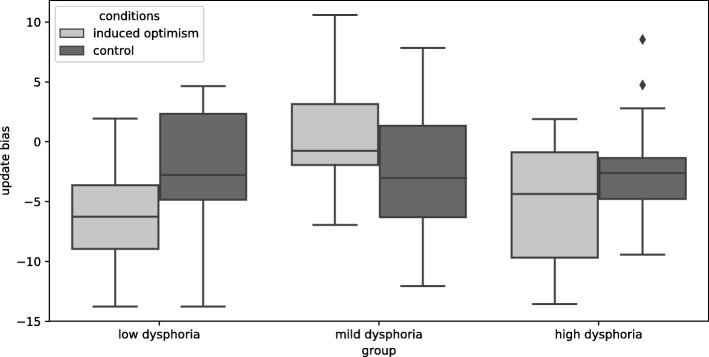
Updating bias(difference between updates for desirable vs. undesirable information) in each group(low, mild, high dysphoria) and condition(induced optimism, control)

### Comparison of response and reaction time to positive and negative events in the inducing optimism manipulation (Table 2)

To examine the effect of the dysphoria level in response to judging positive or negative future events, we conducted mixed two-way ANOVA for the mean response of yes to positive and negative events (dysphoria group as between subject factor and valence of events (positive or negative event) as within subject factor) only to the induced optimism condition (because participants in the control condition did not engage in mental rehearsal manipulation, response data was not collected). A significant interaction was not found (*F* (2, 27 = .98, *p* > .1, partial *η*^*2*^ = .07). The main effect of valence of event was significant (*F* (1, 27) = 156.28, *p* < .001, partial *η*^*2*^ = .85), and participants yes responses to positive events were more frequent with than negative events (*p* < .001, *d* = 3.08). In addition, we conducted a mixed two-way ANOVA for the mean response time to positive and negative events (dysphoria group as the between subject factor and type of events as the within subject factor). Regardless of type of dysphoria group, response times to positive events were faster than negative events (*F* = 58.15, *p* < .001, partial *η*^*2*^ = .68), and the main effect of dysphoria group was not significant (*F* = .228, *p* = .80, partial *η*^*2*^ = .02). In addition, we performed a correlational analysis between the response time to positive or negative events and the updating bias. There were no significant correlations (positive event - updating bias: *r* = .19; negative event – updating bias: *r* = .18). These results indicate that participants in the induced optimism condition were affected by the training regardless of the level of dysphoria.

**Table 2:** Task performance during induced optimism manipulation

Dysphoria group	Response to positive events mean(SD)	Response to negative events mean(SD)	Reaction time (sec) to positive events mean(SD)	Reaction time (sec) to negative events mean(SD)
Low	24.2(2.6) ^**^	8.18(3.8)	22.2(18.7) ^**^	11.7(11.0)
Mild	26.0(3.2) ^**^	12.5(7.3)	19.9(5.9) ^**^	9.3(4.7)
High	23.1(3.8) ^**^	10.8(3.8)	22.3(11.8) ^**^	10.2(2.7)

^**^:*p* < .001

## Discussion

Unrealistic optimism is a biased and often false view of the future, but has significant benefits because it increases well-being and contributes to overall mental health. In the present study, we investigated the effects of induced optimism in individuals with various levels of dysphoria on belief updating when receiving information about the future. We divided participants into three groups (low, mild, high dysphoria) by baseline dysphoria levels, measured by CES-D score, and assigned these groups to the induced optimism or control condition. In addition, participants performed a belief updating task after the induced optimism manipulation There were no differences in dysphoria and optimism between the induced optimism and control conditions. Results showed no effect of induced optimism on both dysphoria and dispositional optimism. Participants in the mild dysphoria group who participated in the induced optimism condition however, showed a high update bias rather than either the high or low dysphoria group. In addition, participants who received the induced optimism training often responded yes to positive rather than negative events, regardless of participant’s degree of dysphoria. These results partially supports the hypothesis that induced optimism would increase the optimistic bias for mild dysphoria. However, contrary to our hypotheses, induced optimism did not change dispositional optimism and depression. Additionally, the low dysphoric group did not show an increased updating bias compared with the mild and high dysphoric groups.

The main finding of the present study was that participants in the mild dysphoria group showed an increased update bias after the induced optimism manipulation. This means increased optimism changed the future prediction of the probability of experiencing adverse life events. That is, optimism might arouse a cognitive process which promotes an underestimation about the probability of risk in participants with mild dysphoria.

These results also suggest that mild dysphoria has a distinctive profile compared to moderate or severe depression. Soderstrom, Davalos, & Vázquez [31] reported that mild depression showed depressive realism but not in moderate depression, and suggested that the level of depression accounts for depressive realism. In addition, contrary to our prediction, results did not indicate the update bias in the low dysphoria group (both the induced optimism condition and control condition), and there were no significant differences in the update bias between the low dysphoria and high dysphoria groups. These data are not consistent with Korn et al. (2014) which demonstrated a differential update bias between patients with depression and healthy participants. This inconsistency arouses suspicion about the validity of the belief updating task which is used in the study. However, previous research has indicated that the Japanese are less optimistic than western people [27, 32]. This cultural difference about optimism might cause no update bias in low dysphoria group. Conversely, there were no changes in the CES-D score after induced optimism. These results were consistent with the report from [20]. Their research also showed no difference in depressive mood in the induced optimism manipulation. Both our study and the study from [20] were limited to one study session. More sustained manipulation and practice might be needed to improve depressive symptoms using induced optimism.

\Additionally, results indicated that participants in the induced optimism condition engaged in a mental rehearsal task regardless of the levels of dysphoria, because participants more respond to positive events than negative events as a whole. This result is consistent with [20]. They reported participants in the induced optimism manipulation more often responded yes to positive than negative events, generally. This finding also means there might be no relationship between performance of the mental rehearsal task and change of update bias in the belief updating task. There are other possibilities regarding this issue. Miranda et al. [20] suggested differences of response time to positive or negative events during the mental rehearsal task, where induced optimism manipulation reflects efficiency of manipulation. We compared group difference of response times to positive or negative events during induced optimism manipulation. There was no significant difference between dysphoria groups and no correlation between response time and updating bias. These results did not support relationships between task performance and changes of updating belief. Therefore, we could not examine cause of increased update bias in mild dysphoria group.

Interestingly, there were no significant changes on the LOT-R score in all dysphoria groups and conditions. A recent meta-analysis [18] has indicated that an intervention for increasing optimism had significant effect sizes if they used the positive and negative expectancy measures rather than the LOT-R. The LOT-R measures generalized optimism and pessimism. However, there was a difference between the eastern and western culture in factor structure of the LOT-R [27]. This means that the LOT-R might be not absolutely appropriate for measuring effects of induced optimism. In the present study, we examined the effect of induced optimism not on the positive and negative expectancy measures but on the belief changes in response to receipt of both desirable and undesirable information about future life events. In many cases, the positive and negative expectancy measures might be an estimation of future events at the present moment but not necessarily reflect a change in estimation from new information (either desirable or undesirable). An optimism intervention study might have assessed optimism using LOT-R [18], but also it may be important to consider the effects of optimism interventions by using updating beliefs about the future as another aspect of optimism.

The present study has some limitations. First, although participants were pre-selected for level of dysphoria, the sample consisted of college undergraduates and was not a clinical sample. The effects of the study should be replicated in a clinical sample. Second, the sample size was small. Sixty-nine participants into three groups for the degree of dysphoria and two conditions. The unequal and small cell sizes may have affected the current results. This affects the power of the study and the subsequent interpretation of the results. Finally, we could not examine the longitudinal effects of induced optimism training because the present study used a single session training. Further research is needed for investigate effects of the intervention on the belief updating task and develop more efficacious interventions which induce optimism over time.

## Conclusions

The current study indicates that imagining a positive future enables mild dysphoric individuals to increase positive beliefs about the future to a certain extent. However, contrary to our prediction and previous study, imagining a positive future did not alter both dysphoria and dispositional optimism. Regardless of this fact, induced optimism provides a potential to cause both an overestimation of desirable information and underestimation of undesirable information followed by the belief updating task. Future research is needed to replicate the current findings with a bigger sample size and include participants with clinical depression.

## Data Availability

The datasets generated and analyzed during the current study will be available from the author upon reasonable request.

## Abbreviations

ANCOVA: Analysis of Covariance
ANOVA: Analysis of Variance
CES-D: Center for Epidemiologic Studies Depression scale
LOT-R: Life Orientation Test Revised
SD: Standard Deviation
